# Jejunal Diverticulosis: An Atypical Lead Point for Small Bowel Volvulus

**DOI:** 10.7759/cureus.56125

**Published:** 2024-03-13

**Authors:** Divij Agarwal, Iqbal Ali, Varun Shetty

**Affiliations:** 1 General Surgery, Dr. D.Y. Patil Medical College, Hospital and Research Centre, Pune, IND

**Keywords:** surgical anastomosis, malrotation, mesenteric ischaemia, mid gut volvulus, small bowel diverticulosis

## Abstract

The occurrence of small intestinal diverticula is relatively infrequent compared to its counterpart in the large bowel. Duodenal diverticula exhibit a significantly higher prevalence within the small intestine compared to jejunoileal diverticula, with a ratio of five to one. The occurrence of jejunoileal diverticula exhibits considerable variability and has been documented to range from 0.5% to 2.3% in radiographic series, while autopsy studies have reported rates as high as 7%. We present the clinical details of a 65-year-old male patient who sought medical attention due to a constellation of symptoms, including abdominal pain, vomiting, and obstipation. After adequate resuscitation with IV fluids and preoperative preparation, the patient was transported to the operating room for an emergency exploratory laparotomy. Multiple jejunal diverticuli were identified in the proximal jejunum at the antimesenteric border, less than three feet from the duodenojejunal (DJ) junction. The terminal ileum was found to be 360° rotated counterclockwise around the small bowel mesentery, causing the small intestine to appear congested; however, after clockwise de-rotation, the small bowel regained its normal color. Adhesiolysis and small bowel decompression were performed, and the patient had an uneventful recovery.

## Introduction

Diverticular disease involving the duodenum and small intestine are relatively common anatomical entities but seldom cause clinical problems. In comparison with its large bowel counterpart, the number of reported incidences of small bowel diverticula is considerably low. Amongst small bowel diverticula, those of duodenal origin supersede jejunoileal diverticula by a vast margin of up to five times [1]. The occurrence of jejunoileal diverticula exhibits considerable variability and has been documented to range from 0.5% to 2.3% in radiographic series, while autopsy studies have reported rates as high as 7% [2,3]. The etiology of these diverticula is known to be multifactorial, encompassing a wide range of contributing factors [3]. Diverticula of the small bowel are classified as either congenital or acquired. Meckel’s diverticulum and intraluminal diverticula represent true congenital diverticula owing to the presence of all bowel wall layers. Acquired diverticula, on the other hand, arise from weaker areas of the bowel wall surrounding the entry points for blood vessels. This anatomical detail allows for the protrusion of mucosa and submucosa alone, thereby forming the basis of the term “false diverticula.” The majority of cases of small bowel diverticulosis remain silent with a relatively indolent course. However, a retrospective analysis of patients reported a notable proportion (10-30%) of diverticular disease exhibiting concomitant complications, including diverticulitis, perforation, bleeding, or obstruction [4]. These complications were found to be associated with a progressive escalation in both morbidity and mortality rates. We present the clinical details of a 65-year-old male patient who sought medical attention due to a constellation of symptoms, including abdominal pain, vomiting, and obstipation, highlighting the diagnostic challenges and management strategies employed.

## Case presentation

**Figure 1:**
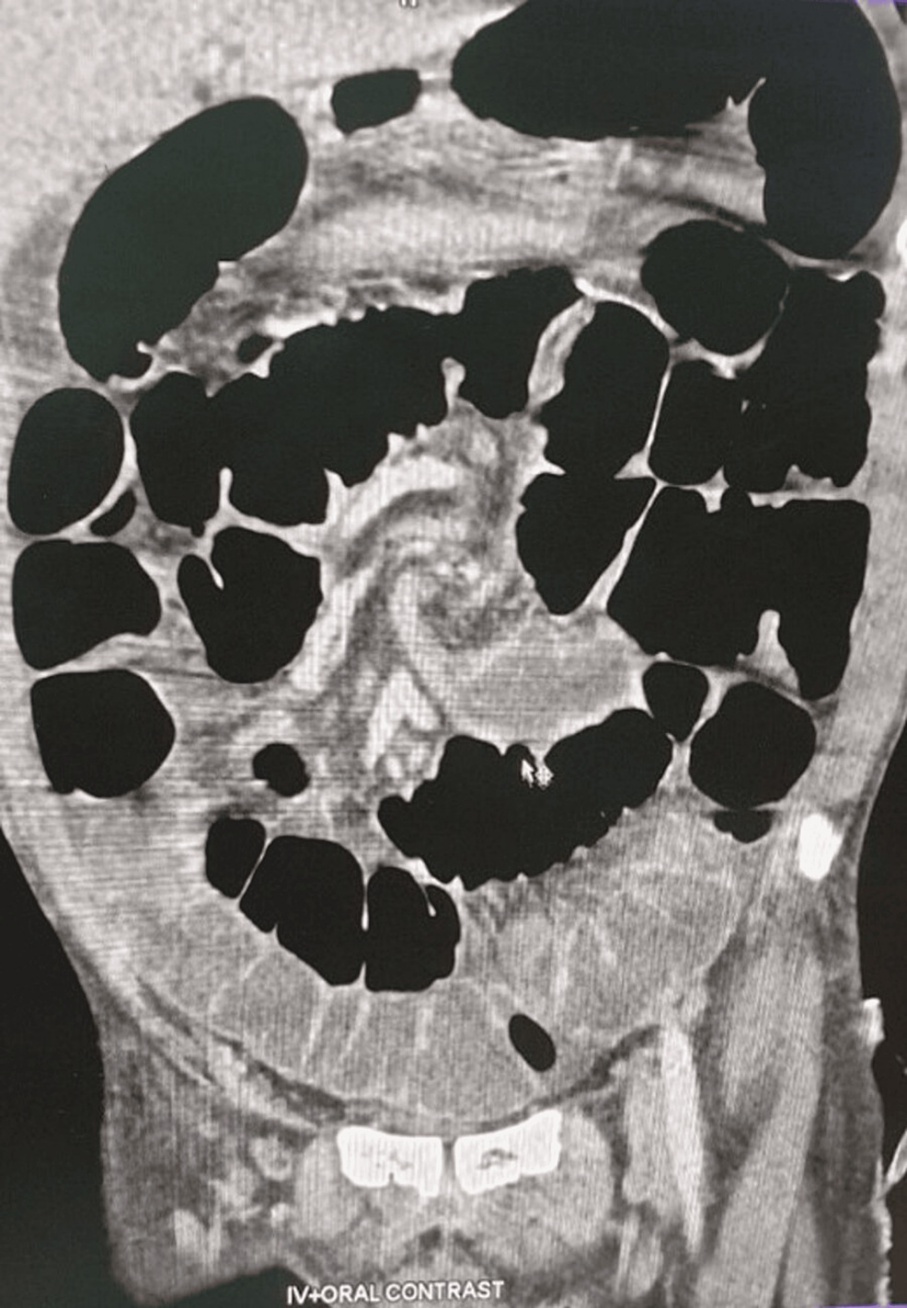
Coronal CECT view showing “whirlpool sign." Dilated ileal loop just proximal to the point of twisting and ileal segment twisting around the mesentery. CECT: Contrast-Enhanced Computed Tomography.

A 65-year-old man presented to the emergency department with chief complaints of abdominal pain for the past four days. The pain was gradual in onset, mild, aching, generalized, and had worsened the day before. Additionally, he experienced multiple episodes of regurgitation, absence of flatus and bowel movements for the past three days, and symptoms of abdominal distension and retching. Ten years ago, the patient experienced a comparable episode of abdominal distention for which he did not seek medical attention and which resolved spontaneously. The patient had an average build and normal vital signs upon examination. Examination of the abdomen revealed generalized abdominal distention and tenderness. There were no symptoms or abdominal signs of peritonitis present. Hyperperistaltic bowel noises were noticed. Laboratory tests were unremarkable. X-ray of the abdomen (erect and supine) showed clumped small bowel loops in the left upper quadrant with multiple air-fluid levels. Transabdominal sonography (TAS) revealed conspicuous bowel loops with minimal inter-bowel-free fluid. The contrast-enhanced CT of the abdomen revealed that the jejunal and ileal loops (proximal and mid-ileal) were dilated to a maximal diameter of 3.7 cm. In the distal ileum, a segment of the ileum was observed to twist around its mesentery by 360° and manifest a whirlpool sign (Figure 1). A preoperative diagnosis of small bowel volvulus was made.

**Figure 2:**
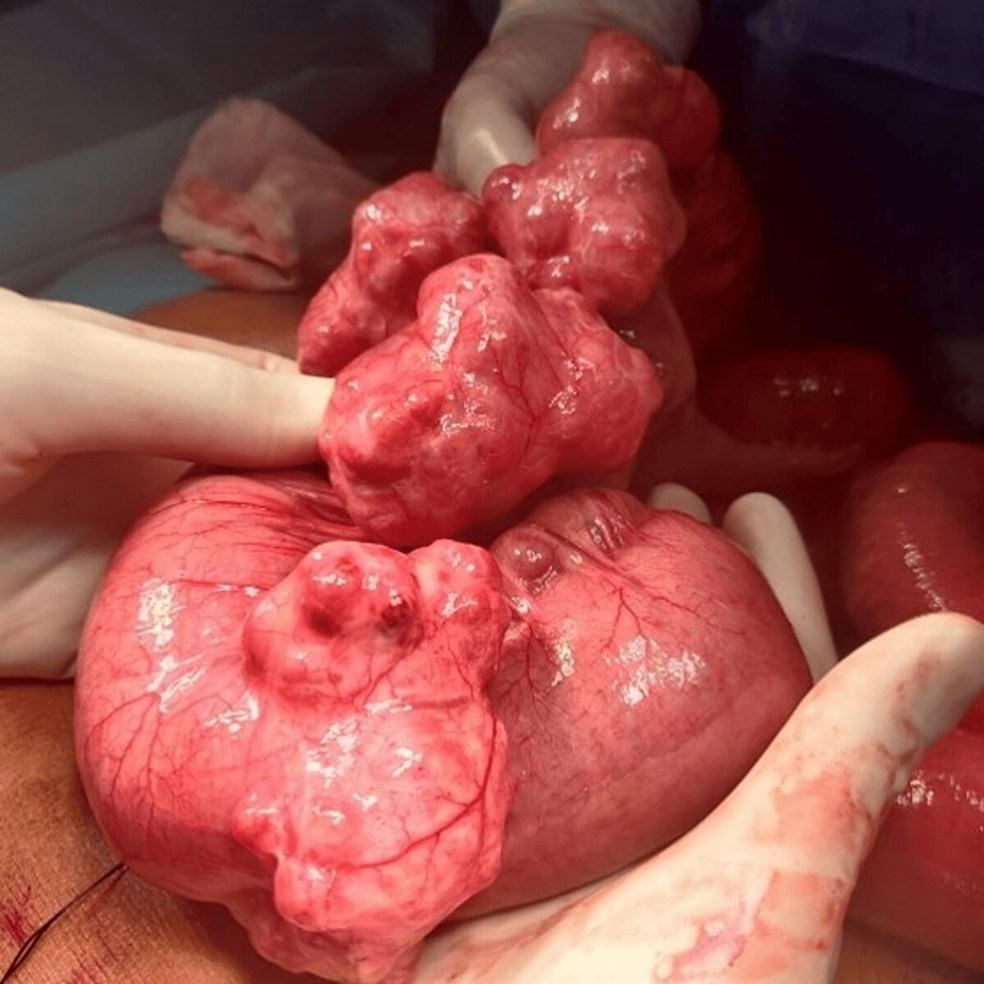
Intra-operative photograph showing multiple diverticula located at anti-mesenteric border of the jejunum.

After adequate resuscitation with IV fluids and preoperative preparation, the patient was transported to the operating room for an emergency exploratory laparotomy. The entire small intestine was discovered to be viable but mildly dilated intraoperatively. A comprehensive examination of the small intestine revealed six large diverticula in the proximal jejunum at the antimesenteric border, less than three feet from the DJ junction (Figure 2).

**Figure 3:**
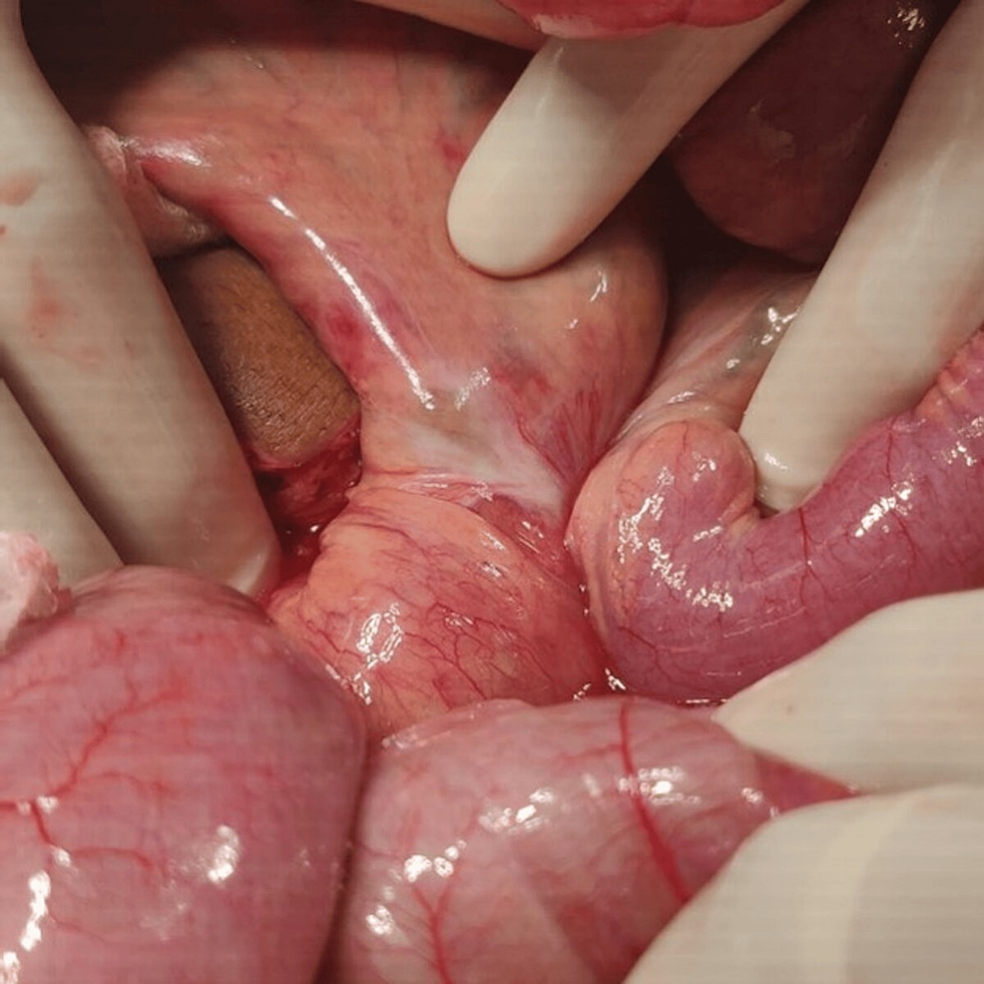
Intra-operative photograph showing distal ileal loop twisting around mesentery and proximal jejunum.

The terminal ileum was found to be 360° rotated counterclockwise around the small bowel mesentery, causing the small intestine to appear congested; however, after clockwise de-rotation, the small bowel regained its normal color (Figure 3).

The caecum and ascending colon were dilated due to a dense fibrous adhesive band compressing the mid-transverse colon, resulting in proximal dilatation. Additionally, numerous adhesions were found between the gallbladder, hepatic flexure of the colon, and duodenum, as well as between the ascending colon and the peritoneum. After performing adhesiolysis, a decision was made to resect the segment of the jejunum bearing the diverticula in question to prevent any recurrence in the future. An end-to-end jejunoileal anastomosis was performed in four layers. The small intestine was reposited into the abdomen, which was then closed in layers. The post-operative period was uneventful. He began an oral diet on the second postoperative day and was discharged on the eighth postoperative day.

## Discussion

Small bowel volvulus, a condition characterized by the twisting of the small intestine and its accompanying mesentery, is a topic of interest in the medical field. An exceptionally uncommon diagnosis in adults, the occurrence of volvulus secondary to jejunal diverticula as a complication is even more infrequent [4]. Jejunoileal diverticula are a rare form of small bowel diverticula, with a prevalence ranging from 0.002% to 5%. The risk of the condition escalates with advancing age, reaching its highest point during the sixth and seventh decades of life. Jejunoileal diverticula are a type of acquired pseudodiverticula that develop as a result of jejunoileal dyskinesia. This condition leads to elevated intraluminal pressures, ultimately causing the herniation of the mucosa and submucosa through the weakest point of the bowel wall's muscularis propria. The mesenteric border, where blood vessels enter the bowel wall, represents the weakest site and is, therefore, the most susceptible to herniation. While the exact etiology remains unclear, several factors have been proposed to contribute to its development. These include age-related changes in bowel motility, chronic inflammation, and structural abnormalities of the small bowel. Clinical manifestations of jejunal diverticula can vary widely, ranging from asymptomatic cases to severe complications such as diverticulitis, bleeding, or perforation. Diagnosis often involves a combination of imaging studies. The presence of these structures can vary, with approximately one-third (33%) being solitary and the remaining two-thirds (66%) occurring in multiple clusters. These clusters can be found predominantly in the jejunum, ranging from 55% to 80% of cases, or in the ileum, accounting for 15% to 38%. In a small percentage of instances, these structures can be present in both the jejunum and ileum, occurring in approximately 5% to 7% of cases [5,6]. Chronic symptoms, such as abdominal pain, nausea, vomiting, flatulence, and irregular bowel habits, are commonly observed but tend to lack specificity. Complications associated with diverticula manifest in approximately 15%-20% of patients, encompassing a range of issues such as hemorrhage, intestinal obstruction, diverticulitis, and perforation.

A small percentage of individuals, ranging from 2% to 4.6%, may experience acute obstruction due to various factors such as adhesions, intussusception, volvulus, and external compression caused by a diverticulum filled with fluid. In rare cases, obstruction can also occur at the diverticulum or the ileocecal valve due to an enterolith formed within the diverticulum [7,8]. Midgut volvulus can be categorized as primary or secondary. Those resulting from diverticula fall under the secondary type category, which can also be attributed to post-operative adhesions and tumors. The potential initiator of the volvulus could be attributed to the involved segment, characterized by its fluid-filled composition and greater weight compared to the non-involved portion [9]. The operative intervention is contingent upon the intraoperative state of the bowel. In instances where the bowel remains viable, a straightforward de-rotation procedure is conducted, rendering fixation unnecessary. Resection of the bowel segment bearing the diverticula is indicated in selected situations, including bowel gangrene, diverticular perforation, diverticular abscess formation, and intestinal obstruction. In addition, cases with recurrent intestinal obstruction owing to adhesions forming between the diverticula and surrounding structures also warrant surgical resection and anastomosis. In cases involving a gangrenous bowel segment, two possible approaches can be considered: resection and primary anastomosis or the creation of an enterostomy. According to follow-up studies, the recurrence of small bowel volvulus has not been observed in patients who have undergone de-rotation without additional interventions [10,11].

## Conclusions

Management of jejunal diverticulosis poses a unique set of challenges for healthcare professionals. Managing this condition requires careful consideration of several factors to ensure optimal patient outcomes. Owing to the extensive array of presentations and a wide plethora of management options, jejunal diverticulosis should remain a potential though rare cause of small bowel obstruction, thus facilitating appropriate management and reducing associated morbidity and mortality.
